# Local and Systemic Concentrations of Pattern Recognition Receptors of the Lectin Pathway of Complement in a Cohort of Patients With Interstitial Lung Diseases

**DOI:** 10.3389/fimmu.2020.562564

**Published:** 2020-09-23

**Authors:** Severin Vogt, Marten Trendelenburg, Michael Tamm, Daiana Stolz, Katrin Esther Hostettler, Michael Osthoff

**Affiliations:** ^1^Division of Internal Medicine, University Hospital of Basel, Basel, Switzerland; ^2^Department of Biomedicine, University of Basel, Basel, Switzerland; ^3^Department of Clinical Research, University of Basel, Basel, Switzerland; ^4^Clinic of Respiratory Medicine, University Hospital of Basel, Basel, Switzerland

**Keywords:** complement system, mannose-binding lectin, interstitial lung disease, sarcoidosis, idiopathic pulmonary fibrosis, ficolin-2, ficolin-3, bronchoalveolar lavage fluid

## Abstract

**Background:**

The role of the lectin pathway of complement in the pathogenesis of interstitial lung diseases (ILDs) is largely unknown. Pattern recognition receptors (PRR) of the lectin pathway are involved in the clearance of apoptotic cells either via activation of the complement system or as direct opsonins. As recent findings suggest a role of apoptosis in the development of pulmonary fibrosis, the influence of plasma lectins has lately been considered in various ILDs, but data on local concentrations in the lungs are lacking. This study investigated the role of mannose-binding lectin (MBL), ficolin-2 and ficolin-3 in ILD patients with a focus on idiopathic pulmonary fibrosis (IPF) and sarcoidosis.

**Methods:**

A case control study was conducted involving 80 patients with different forms of ILD as well as 40 control patients undergoing routine flexible bronchoscopy with bronchoalveolar lavage (BAL). Plasma and BAL fluid (BALF) levels of MBL, ficolin-2 and ficolin-3 as well as complement split products C4d and C5a (only in BALF) were measured by enzyme-linked immunosorbent assays. Eight single-nucleotide polymorphisms (SNPs) of MBL and ficolin-2 were determined by genotyping and tested for their association with ILDs.

**Results:**

We included 35, 35, 10, and 40 patients with sarcoidosis, idiopathic pulmonary fibrosis (IPF), other ILD, and a control group, respectively. BALF but not plasma levels of the three PRR were significantly elevated in sarcoidosis patients compared to a control group without ILD (MBL: median 66.8 vs. 24.6 ng/ml, *p* = 0.02, ficolin-2: 140 vs. 58.8 ng/ml, *p* = 0.01, ficolin-3: 2523 vs. 1180 ng/ml, *p* = 0.02), whereas the frequency of the investigated SNPs was similar. In line, complement split products were markedly elevated in BALF of sarcoidosis patients (C4d, median 97.4 vs. 0 ng/ml, *p* = 0.10; C5a, 23.9 vs. 9.1 ng/ml, *p* = 0.01). There was a weak positive correlation of BALF ficolin-3 with serum neopterin, a marker of sarcoidosis activity. In IPF patients, we observed numerically higher MBL plasma and BALF levels (plasma, median 1511 vs. 879 ng/ml, *p* = 0.44; BALF, 37.5 vs. 24.6 ng/ml, *p* = 0.7) as well as lower ficolin-2 plasma levels (plasma 1111 vs. 1647 ng/ml, *p* = 0.11). Ficolin-2 plasma levels were inversely correlated with the forced vital capacity (*r* = 0.55, *p* = 0.1).

**Conclusion:**

This is the first study to simultaneously assess systemic and local lectin pathway protein levels in ILD patients. Our data suggest an involvement of PRR of the lectin pathway in the pathogenesis of sarcoidosis given the significantly higher BALF levels compared to a control group. Additional analyses in a larger patient cohort are required to confirm or refute a potential effect of local and/or systemic ficolin-2 levels in IPF patients.

## Introduction

The term interstitial lung disease (ILD) comprises a diverse family of diffuse parenchymal lung disorders of both known and unknown causes. They are classified into four major categories: ILDs of known association (e.g., drugs, connective tissue diseases), granulomatous ILDs (e.g., sarcoidosis), idiopathic interstitial pneumonias and rare ILDs (1). Despite its different etiologies, some ILDs share common pathophysiological characteristics such as an increased proliferation of fibroblasts and myofibroblasts and accumulation of extracellular matrix, subsequently leading to interstitial pulmonary fibrosis (2). Previous findings suggest a key role of apoptosis in the development of pulmonary fibrosis. Increased epithelial cell apoptosis as well as decreased apoptosis of myofibroblasts have consistently been found in the lung tissue of IPF patients and likely contribute to the fibrotic process (3). Several murine models support this hypothesis, showing that the inhibition of epithelial apoptosis ameliorates bleomycin-induced lung fibrosis (4, 5). Moreover, it has been proposed that an impaired clearance of apoptotic material due to defective efferocytosis might maintain an inflammatory state and affect alveolar epithelial repair (6).

The role of the complement system in the clearance of apoptotic cells is well established (7, 8). One of the three pathways that can activate the complement system is the lectin pathway. Six distinct initiators of the lectin pathway have been described in humans: Mannose-binding lectin (MBL), Collectin-10 and 11, ficolin-1 (M-ficolin), ficolin-2 (L-ficolin) and ficolin-3 (H-ficolin) (9). These proteins are soluble pattern recognition molecules that recognize so-called pathogen-associated molecular patterns (PAMPs) on the surface of pathogens, apoptotic and necrotic cells. The binding to PAMPs leads to a conformational change of the associated serine proteases MBL associated protease-1/2, which enables the cleavage of C4 and C2, thus initiating the complement cascade. Lectins are also able to serve as direct opsonins, and the opsonization of cells or pathogens leads to enhanced phagocytosis by macrophages and dendritic cells. This process is also referred to as an immunologically silent phagocytosis, because no activation of the complement system occurs (10, 11).

In the context of ILD, some evidence points to a relevant involvement of the lectin pathway. A case control study found a link between MBL deficiency and early onset IPF and familial cases (12). Similarly, MBL deficiency was associated with coal workers’ pneumoconiosis, implying an important role of MBL in pulmonary host defense (13). Ficolin-2 plasma levels above the median were shown to be predictive for a longer progression-free survival in IPF (14). On the other hand, two studies found an association between high ficolin-2 and MBL plasma levels and systemic sclerosis associated ILD (SSc-ILD), which was supported by a higher frequency of wild-type *FCN2* and *MBL2* genotypes in SSc-ILD patients (15, 16). The authors hypothesized that recurrent lectin-mediated ischemic reperfusion injuries might have induced an endothelial dysfunction and enhanced endothelial and epithelial apoptosis in the lung tissue, thus triggering a fibrotic response. Regarding ficolin-3, low plasma levels were related to the development of SSc-ILD and sarcoidosis, respectively, indicating that an impaired clearance of apoptotic cells due to decreased ficolin-3 levels might play an important role (17, 18).

However, these studies focused on the plasma levels of lectins only, whereas data about lung concentrations, reflecting the site of the disease, are lacking. Moreover, to our knowledge to date no studies exist concerning a broader spectrum of ILDs. We hypothesized that such information could further enlighten the role of lectins in the complex pathogenesis of ILD and particularly IPF. We therefore measured lectin concentrations in both plasma and BAL fluid, determined selected MBL and ficolin-2 polymorphisms and assessed their association to ILD.

## Materials and Methods

We conducted an unmatched case-control study at the University Hospital of Basel, Basel, Switzerland, from August 2017 until March 2019, involving 80 consecutive patients with interstitial lung diseases. We classified these patients into three distinct groups: IPF, ILD other than IPF or sarcoidosis (ILD-O) and sarcoidosis. We compared their lectin concentrations in ethylenediaminetetraacetate (EDTA) plasma and bronchoalveolar lavage fluid (BALF) and the presence of selected polymorphisms to a heterogeneous group of 40 patients with no evidence of interstitial lung disease. The Ethical Review Board of the University Hospital of Basel approved the sample collection (EKBB 05/06). Written informed consent was obtained from all included patients undergoing routine diagnostic bronchoscopy at the University Hospital of Basel, Basel, Switzerland.

### Inclusion Criteria

All patients were over 18 years old. The distinct interstitial lung diseases were classified based on clinical, radiologic, serologic and histopathological features. In the case of IPF, the diagnosis was made according to the guidelines of the American Thoracic Society (19). For the diagnosis of sarcoidosis, characteristic clinical and radiologic features were required and had to be confirmed by characteristic histopathology findings. All cases were discussed at an multidisciplinary board for interstitial lung diseases (Respiratory Medicine, Pathology, and Radiology, University Hospital of Basel). Patients of the control group had various conditions that warranted a bronchoalveolar lavage, but had no suspicion of an ILD. Patients with clinical evidence of an acute infection were excluded.

### Samples

From each patient blood samples were collected in EDTA-tubes prior to the bronchoscopy. Bronchoscopies were performed according to standard procedures. For the bronchoalveolar lavage, three 50 ml aliquots of a sterile, 0.9% NaCl solution were infused through the aspiration port of the bronchoscope. BALF was then obtained by immediately suctioning back the instilled fluid. After obtaining an aliquot of non-centrifuged BALF, EDTA, and BALF tubes were centrifuged (10 min, 3000 rpm), and whole- blood-, plasma- and cell-free BALF samples were collected in tubes. All tubes were stored at −80°C for subsequent analysis. We collected clinical characteristics (baseline characteristics, comorbidities, medication etc.) from patient records of the clinical information system of our hospital.

### Measurements

We quantified MBL levels with enzyme-linked immunosorbent assays, as previously described (20). In brief, we used microtiter plates (Nunc Maxisorp, Thermo Fisher Scientific, Basel, Switzerland), which we coated with a 1 mg/ml mannan stock solution (Sigma M7504, Buchs SG, Switzerland). 1x TBS buffer (used for incubation of samples and subsequent antibody/labeling steps) was prepared from a 10x TBS stock solution, which contains 80 g sodium chloride, 2 g potassium chloride and 60.5 g TRIS Base filled up to 1000 ml with distilled water. Subsequently, 1.1 g calcium chloride and 0.25 ml Tween 20 (Sigma P9416, Buchs SG, Switzerland) was added per 500 ml 1x TBS buffer. After coating, the samples were diluted in TBS and then incubated in duplicates for 90 min. Subsequently, the plates were incubated with a biotinylated mouse-antihuman MBL antibody (HYB131-01B, Bioporto Diagnostics, Denmark) followed by the addition of Extravidin Peroxidase (Sigma E2886). Afterward, TBM substrate solutions (BD OptEIA, BD Biosciences, Allschwil, Switzerland) were applied to develop the plates., Then, 0.5 M H_2_SO_4_ was added to stop the reaction and the optical density was read instantly at 450 nm on a microplate reader (Synergy H1, BioTek, Sursee, Switzerland).

For each assay, a standard dilution series was evaluated, using pooled human serum with a known MBL-concentration (BioPorto Diagnostics, Denmark). A standard curve was then calculated in order to estimate the MBL-concentration of the samples. This estimate was then multiplied by the corresponding dilution factor. Samples with an absorbance above the absorbance of the highest standard were repeated at a higher dilution.

Ficolin-2, ficolin-3, Urea, complement split product C4d and complement split product C5a levels were measured similarly with commercially available assay kits (Ficolin-2: Abcam, Cambridge, Great Britain, ficolin-3: Hycult Biotech, Uden, Netherlands, Urea and C5a: Thermo Fisher, Basel, Switzerland, C4d: Svar Life Science, Malmö, Sweden), following the instructions of the corresponding manuals.

The dilution for plasma samples was as follows: 1:100 for MBL, 1:100 for ficolin-2, 1:300 for ficolin-3 and 1:20 for Urea. Samples with a high absorbance were measured again at a higher dilution (1:400 for MBL, 1:300 for ficolin-2). BALF samples were diluted 1:2 for MBL, ficolin-2, ficolin-3, C4d and C5a, and 1:1 for Urea. Lectin and complement split product concentrations in BALF were measured using centrifuged BALF whereas for urea, non-centrifuged BALF was used.

### Estimation of BALF Solute Concentration

The aspirated BALF is diluted by a varying amount of saline instillation during the procedure. In order to obtain a reliable estimate of the volume of the endogenous epithelial lining fluid (ELF), the measurement of urea has been proposed (21). The major advantage of urea is that it diffuses freely through the alveolar wall (22) and as such, the concentration of urea in plasma and ELF can be assumed to be the same. Following this assumption, the volume of ELF and ELF solute concentrations can be calculated (formulas provided in the Supplementary Material). For the purpose of statistical analysis, samples with absorbance values below half of the absorbance of the lowest standard were set to 1/4 of the concentration of the lowest standard.

### MBL2 and FCN2 Genotyping

We genotyped *MBL2* and *FCN2* single nucleotide polymorphisms (SNPs) by allele specific polymerase chain reaction (PCR), in order to determine allelic variants of *MBL2* and *FCN2*. Briefly, whole blood samples were lysed with a lysis solution and a stabilizer (Applied Biosystems, Foster City, United States) was added. For DNA amplification, 1 μl of this DNA lysate and 4 μl of a genotyping assay mix, consisting of 0.25 μl TaqMan probe, 2.5 μl TaqMan GTXpress Master Mix (Applied Biosystems, Foster City, United States) and 1.25 μl DNase-free water, were applied on a 384-well plate. The PCR was performed in a ViiA7 thermocycler (Applied Biosystems, Foster City, United States) according to the manufacturer’s instructions. After the amplification, allelic variants were determined using the software provided by ViiA7. For *MBL2*, we chose four SNPs known to have a considerable impact on MBL serum levels of which rs1800450 (A/B), rs1800451 (A/C) and rs5030737 (A/D) are located on exon 1 and rs7096206 (Y/X) on the promoter region. We classified the resulting *MBL2* genotypes into established categories (23) as low (0/0, XA/0), intermediate (XA/XA, YA/0) and high producing (YA/XA, YA/YA), where exon 1 mutations are denoted as 0 (wild-type alleles as A), and the promoter mutation as X (wild-type allele as Y). For *FCN2*, common SNPs of the promoter (−602G > A; rs3124953, and −4A > G; ss32469537) and exon 8 (+6424G > T; rs7851696, +6359C > T; ss32469544) were determined (24).

### Statistical Analysis

All statistical analysis were performed with R (version 3.4.1). The Mann–Whitney *U* test was used to compare the lectin levels of the cases to the control group. Continuous variables were correlated with spearman correlation. For categorical variables we used the chi-squared test or fisher exact test as appropriate. All test were performed two sided at a 5% alpha level (=Type 1 error rate).

## Results

### Baseline Characteristics and Classification of Controls and Cases

A total of 120 patients were included in the study and classified into four groups: IPF (10 patients), ILD other than IPF and sarcoidosis (ILD-O, 35 patients), sarcoidosis (35 patients) and a control group (40 patients). The ILD-O group comprised a wide range of ILDs classified as follows: hypersensitivity pneumonitis (*n* = 12), connective tissue disease-associated ILD (*n* = 6), unclassified ILD (*n* = 10), respiratory bronchiolitis- associated interstitial lung disease (*n* = 3) and Langerhans cell histiocytosis (*n* = 2). The control group consisted of patients with a variety of diseases including stable COPD (*n* = 11), advanced pulmonary cancer (*n* = 12) and pulmonary nodules or lymphadenopathy of unclear etiology (*n* = 5).

Table 1 provides a summary of baseline characteristics of the four groups. Mean age [standard deviation (SD)] was 63 (14), 77 (11), 66 (13), and 53 (13) years in the control, IPF, non-IPF and sarcoidosis group, respectively. Gender was equally distributed with 17 (42%), 5 (50%), 16 (46%), and 14 (40%) females in the respective groups. Inflammatory markers were normal or only mildly elevated in the majority of patients.

**TABLE 1:** Baseline characteristics of cases and controls.

	**Controls**	**IPF**	**ILD-O**	**Sarcoidosis**
Number of patients	40	10	35	35
Age, mean (SD)	63 (14)	77 (11)	66 (13)	53 (13)
Female gender, *n* (%)	17 (42)	5 (50)	16 (46)	14 (40)
Pack years, mean (SD)	36 (35)	22 (23)	20 (28)	7 (10)
DM type 2, *n* (%)	3 (7)	2 (20)	6 (17)	4 (11)
Newly diagnosed, *n* (%)*	29 (72)	5 (50)	25 (71)	32 (91)
**Smoking History, *n* (%)**				
Never	7 (18)	2 (20)	17 (49)	14 (40)
Active	13 (32)	2 (20)	8 (23)	6 (17)
Previous	12 (30)	5 (50)	9 (26)	9 (26)
NA	8 (20)	0 (0)	1 (3)	6 (17)
C-reactive protein, median (IQR)	3 (9)	6 (4)	7 (8)	3.3 (6.8)
**Immunosuppression before BAL, *n* (%)**				
Yes	0 (0)	0 (0)	10 (29)	2 (6)
No	40 (100)	10 (100)	25 (71)	30 (86)
unknown	0 (0)	0 (0)	0 (0)	3 (9)
**Lung function parameters, mean (SD)**				
TLC, % predicted	–	81 (14)	80 (17)	–
FVC, % predicted	–	88 (17)	84 (27)	–
FEV1, % predicted	–	93 (18)	85 (24)	–
FEV1/VCmax	–	77 (14)	77 (17)	–
DLCOc, % predicted	–	65 (23)	58 (19)	–

BAL, bronchoalveolar lavage; SD, standard deviation; DM type 2, Diabetes mellitus type 2; TLC, total lung capacity; FVC, forced vital capacity; FEV1, forced expiratory volume in 1 s; VCmax, vital capacity; DLCOc, diffusing capacity of the lung for carbon monoxide (adjusted for hemoglobin); IQR, interquartile range. *Percentage of patients with a diagnosis of sarcoidosis, IPF, ILD-O (or other diagnoses in the control group) established at the time of bronchoscopy with BALF collection.

### Lectin Protein Plasma Levels of Cases and Controls

**FIGURE 1:**
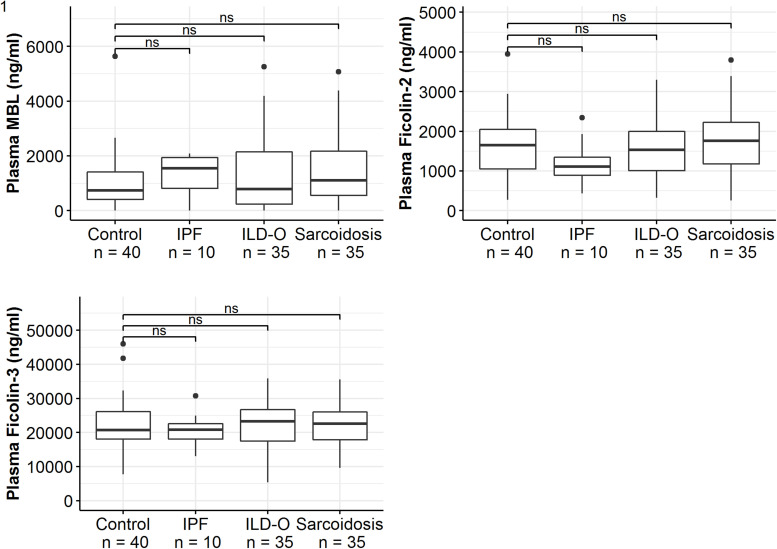
Mannose-binding lectin, ficolin-2 and ficolin-3 plasma concentrations of idiopathic pulmonary fibrosis (IPF), non-IPF patients other than sarcoidosis (ILD-O), sarcoidosis and control patients. Horizontal bars annotated with “ns” denote non-significant comparisons (Mann–Whitney *U* test).

Median MBL plasma levels were markedly elevated in the IPF and sarcoidosis group when compared to the control group, although the difference was not statistically significant (Table 2 and Figure 1, IPF, median 1511 ng/ml, *p* = 0.44; Sarcoidosis 1111 ng/ml, *p* = 0.11; Control 879 ng/ml). Regarding ficolin-2, IPF patients had moderately lower plasma levels than the control group (median 1111 vs. 1647 ng/ml, *p* = 0.11), though the difference was not statistically significant.

**TABLE 2:** Lectin plasma and BALF levels of cases and controls.

	**Controls**	**IPF**	**ILD-O**	**Sarcoidosis**
**Lectin plasma levels, median (IQR)**				
MBL, ng/ml	879 (1312)	1511 (1117)	786 (1914)	1111 (1672)
Ficolin-2, ng/ml	1647 (993)	1111 (454)	1529 (991)	1763 (1047)
Ficolin-3, ng/ml	20649 (8017)	20786 (4534)	23266 (9207)	22612 (8189)
**Lectin and complement split product BALF levels, median (IQR)**				
MBL, ng/ml	24.6 (73.1)	37.5 (91.9)	38.9 (62.1)	66.8 (124)*
Ficolin-2, ng/ml	58.8 (117)	61.9 (76.2)	45.3 (149)	140 (220)*
Ficolin-3, ng/ml	1180 (2432)	720 (1240)	1075 (3090)	2523 (2398)*
C4d, ng/ml	0 (200)	–	–	97.4 (546)
C5a, ng/ml	9.1 (26.4)	–	–	23.9 (22.6)*

*p-value < 0.05 compared to control group. IQR, interquartile range; BALF, bronchoalveolar lavage fluid; MBL, mannose-binding lectin.

### Lectin and Complement Protein Levels in the Alveolar Compartment

**FIGURE 2:**
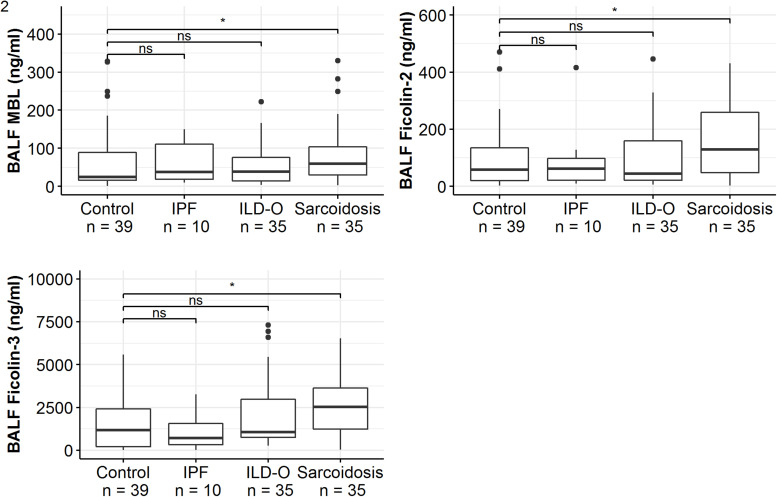
BALF lectin concentrations of idiopathic pulmonary fibrosis (IPF), non-IPF patients other sarcoidosis (ILD-O), sarcoidosis and control patients. Horizontal bars with a star denote statistically significant comparisons (*p*-value < 0.05) whereas bars annotated with “ns” denote non-significant comparisons (Mann–Whitney *U* test).

**FIGURE 3:**
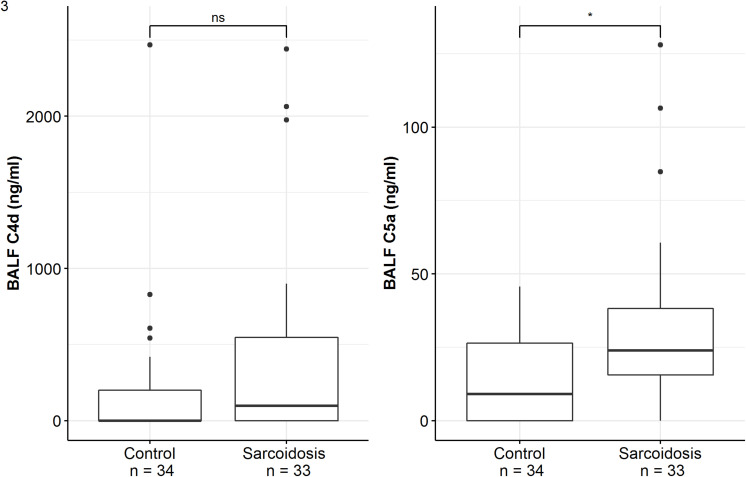
BALF complement split products concentrations of sarcoidosis and control patients. Horizontal bars with a star denote statistically significant comparisons (*p*-value < 0.05) whereas bars annotated with “ns” denote non-significant comparisons (Mann–Whitney *U* test).

Patients of the sarcoidosis group had significantly higher MBL-, ficolin-2 and ficolin-3 BALF levels compared to the controls (Table 2 and Figure 2, MBL, median 66.8 vs. 24.6 ng/ml, *p* = 0.02; ficolin-2 140 vs. 58.8 ng/ml, *p* = 0.01, ficolin-3 2523 vs. 1180 ng/ml, *p* = 0.02). Furthermore, complement split products C4d and C5a were elevated as well in the sarcoidosis group (Table 2 and Figure 3, C4d, median 97.4 vs. 0 ng/ml, *p* = 0.10; C5a, 23.9 vs. 9.1 ng/ml, *p* = 0.01). In contrast, ficolin- 3 BALF levels of the IPF group were slightly lower than the control group’s BALF levels, though the comparison was not statistically significant.

Overall, lectin plasma- and BALF levels were moderately correlated. Correlation of plasma and BALF concentrations in case of MBL and ficolin-2 was stronger (MBL, *r* = 0.47, *p* < 0.01; ficolin-2, *r* = 0.36, *p* < 0.01) compared to ficolin-3 (*r* = 0.2, *p* = 0.03). Importantly, the correlation of lectin protein and complement split product levels in BALF was moderate to strong (C4d; MBL, *r* = 0.38, *p* < 0.01, ficolin-2, *r* = 0.23, *p* = 0.04, ficolin-3, *r* = 0.51, *p* < 0.01, C5a; MBL, *r* = 0.43, *p* < 0.01, ficolin-2, *r* = 0.49, *p* < 0.01, ficolin-3, *r* = 0.72, *p* < 0.01).

### Association of Lung Function Parameters With Lectin Concentrations

Regarding the IPF cohort, higher ficolin-2 plasma levels were moderately correlated with a lower FVC (Table 3, *r* = −0.55, *p* = 0.10) and FEV1 (*r* = −0.48, *p* = 0.16). Whereas ficolin-3 plasma levels also inversely correlated with lung function parameters (FVC, FEV1 and DLCOc) in IPF patients, MBL levels (plasma and BALF) were positively correlated, although none of these correlations were statistically significant (Table 3). In the ILD-O cohort, lower plasma ficolin-2 levels moderately correlated with a worse FVC (Supplementary Table S1, *r* = 0.39, *p* = 0.02).

**TABLE 3:** Spearman correlation between lung function parameters and lectin concentrations in the IPF group.

	**FVC**	**FEV1**	**DLCOc**
	**Spearman’s rho (*P*-value)**	**Spearman’s rho (*P*-value)**	**Spearman’s rho (*P*-value)**
**MBL**			
Plasma	0.50 (0.14)	0.49 (0.15)	0.52 (0.16)
BALF	0.16 (0.66)	0.38 (0.28)	0.38 (0.31)
**Ficolin-2**			
Plasma	−0.55 (0.10)	−0.48 (0.16)	0.13 (0.74)
BALF	−0.41 (0.25)	−0.19 (0.59)	−0.10 (0.81)
**Ficolin-3**			
Plasma	−0.25 (0.49)	−0.39 (0.27)	−0.43 (0.25)
BALF	−0.18 (0.63)	−0.41 (0.24)	−0.1 (0.81)

*MBL, mannose-binding lectin; BALF, bronchoalveolar lavage fluid; FVC, forced vital capacity, FEV1, forced expiratory volume in 1 s; DLCOc, diffusing capacity of the lung for carbon monoxide (adjusted for hemoglobin).*

In the sarcoidosis cohort, the vast majority of patients had normal lung function values, since they were included at the time of diagnosis and thus at a very early stage of the disease. Hence, we did not correlate their lung function values with lectin concentrations.

### Distribution of Genetic Polymorphisms in Cases and Controls

The distribution of *MBL2* polymorphisms was similar in the control, ILD-O and sarcoidosis group. Interestingly, IPF patients had a lower frequency of B and D allelic variants whereas the X variant was present substantially more often compared to the control group. Moreover, high producing *MBL2* genotypes were substantially more frequent in the IPF group (Supplementary Table S2 and Table 4), which is in line with the observed higher median MBL plasma concentrations. Regarding *FCN2*, the allelic variants *FCN2* −4 and *FCN2* + 6359 were more frequent in the IPF group. However, none of the mentioned imbalances between groups were statistically significant (Supplementary Tables S2, S3).

**TABLE 4:** Distribution of *MBL2* genotypes in cases and controls.

	**Controls**	**IPF**	**ILD-O**	**Sarcoidosis**
*MBL2* genotypes, *n* (%)				
High producing	16 (40)	8 (80)	16 (44)	17 (48)
Intermediate producing	17 (42)	1 (10)	9 (26)	15 (43)
Low producing	7 (18)	1 (10)	10 (28)	3 (9)
*P*-value*	Reference	0.21	0.39	0.43

*Fisher exact test.

### Association Between Lectin Complement Protein Plasma and BALF Levels and Sarcoidosis Serum Activity Markers

Lectin plasma levels were not associated with sarcoidosis serum activity markers (ACE, IL2-receptor, neopterin). However, ficolin-3 BALF levels moderately correlated with neopterin (*r* = 0.39, *p* = 0.05).

## Discussion

We intended to further explore a potential association between the lectin pathway and interstitial lung diseases by measuring lectin levels in the plasma as well as for the first time in the BALF in three defined ILD subgroups, i.e., IPF, non-IPF ILD other than sarcoidosis (ILD-O) and sarcoidosis compared to a control group.

Sarcoidosis is a systemic granulomatous disorder of unknown etiology with frequent organ involvement of the lungs. Interestingly, lectin BALF levels but not plasma levels were elevated in the sarcoidosis group, indicating an active role of lectins in the local disease process either as a consequence or as a driver of local inflammation. Importantly, most patients lacked evidence of systemic inflammation. This is noteworthy, as with the exception of ficolin-3, the analyzed lectin pathway proteins are mainly produced in the liver and not locally secreted in the lungs. Hence, elevated lectin BALF levels do not reflect differences in systemic lectin levels (and hence only diffusion), but probably selective direction of lectin pathway proteins to the lungs as part of the disease process. In line, ficolin-3 BALF (but not serum levels) correlated with neopterin, an activity marker of sarcoidosis. Previously, local complement activation and synthesis of downstream complement proteins by alveolar macrophages were demonstrated in patients with sarcoidosis (25, 26). We could confirm this finding, since C4d as well as C5a levels were markedly elevated in BALF of sarcoidosis patients. Pattern recognition molecules of the lectin system may trigger the observed complement activation and thus lead to a sustained pulmonary inflammation. Our data supports this hypothesis, considering the strong correlation between lectin pathway proteins and complement split products. Intriguingly, ficolin-3 was most strongly associated with C4d and C5a. This observation is interesting, as ficolin-3 is the only lectin protein produced locally in the lungs. Hence, a local upregulation during an active pulmonary process is plausible. Another mechanism could be that lectin proteins might attract alveolar macrophages and other phagocytes through their function as opsonins, contributing to the well described mononuclear alveolitis in sarcoidosis (27, 28). Interestingly, a recent case-control study found a link between low ficolin-3 plasma levels and sarcoidosis (18). They suggested that ficolin-3 deficiency might lead to an impaired clearance of late apoptotic cells, cell detritus and environmental antigens, favoring a sarcoid response. We could not confirm this observation. On the contrary, ficolin-3 plasma levels were similar or even higher in sarcoidosis patients compared to our control group, and BALF levels were significantly higher in the present study, which does not favor a link between lectin pathway levels in the lung and impaired local clearance of apoptotic cells in sarcoidosis. Of note, our sample size was smaller and there were important differences in the control group used in the two studies that may be responsible for the difference observed.

In line with previous studies (18, 29), MBL plasma levels of the sarcoidosis group were similar compared to a control group. Future studies should investigate the source of the analyzed lectin proteins (e.g., alveolar macrophages) and their function (e.g., activation of the lectin pathway as a consequence of an underlying infectious agent). In this context, it may be interesting to examine BALF longitudinally during immunosuppressive therapy and assess the severity of disease and the success of treatment in relationship to the initial and follow-up BALF lectin protein levels.

Although not significant, we found differences in lectin protein concentrations in the IPF group, with higher median plasma MBL levels in the IPF group compared to the control or ILD-O group. Moreover, IPF patients had lower ficolin-2 plasma levels. The role of lectins in IPF is largely unknown. A previous study reported an association between MBL deficiency in serum and the development of IPF in young (<55 years) and familial cases of IPF, but not in older, typical IPF patients (12). The patients of our IPF cohort (mean age of 77) had rather elevated than deficient plasma MBL levels, thus confirming that MBL deficiency is probably not implicated in IPF. The elevated MBL plasma levels observed in our study are in line with a higher percentage of high producing MBL genotypes, which suggests that the MBL plasma levels of our IPF patients are not influenced by a disease related process or by inflammation. Similarly, MBL BALF levels were numerically higher in the IPF group compared to the control group. A previous study, which assessed the association of serum MBL levels with SSc-ILD, found a similar association (15). The authors hypothesized, that recurrent lectin-mediated vascular injuries might induce an endothelial dysfunction, leading to pulmonary inflammation and fibrosis. In line, the presence of a vasculopathy in IPF patients has been confirmed by observational studies (30, 31). However, it remains unclear to what extent injured endothelial cells contribute to fibrotic processes in IPF, as current models of IPF consider rather disturbed epithelial-mesenchymal interactions as crucial (32).

Ficolin-2 plasma and BALF levels were negatively correlated with FVC. Interestingly, ficolin-2 plasma levels were also negatively correlated with FVC in patients with SSc-ILD in previous studies (15, 16). Conversely, a previous study reported an association between ficolin-2 plasma levels above the median and progression free survival in IPF, suggesting a protective effect of high ficolin-2 plasma levels (14).

The median lectin plasma and BALF concentrations of the ILD-O group did not differ meaningfully from the control group, implying no relevant role of lectins in interstitial lung diseases other than IPF. However, the ILD-O group comprised a variety of ILDs with differing pathophysiological characteristics, thus subgroup effects could have been masked.

The main limitation of the study is the small sample size and the multiple statistical comparisons performed, potentially inflating type-I error. This is in particular relevant for the IPF group, which only contained 10 patients. Moreover, the non-IPF group contains various ILDs with various causes and disease processes. It is possible that subgroups differ in relation to lectin involvement. Unfortunately, our sample size was too low for a subgroup analysis. Further, the control group comprised patients with a variety of lung diseases, and we lack a control group of healthy individuals. Although none of these patients had signs of interstitial lung changes, the presence of another lung disease might have influenced lectin levels.

## Conclusion

In conclusion, the present study is the first to simultaneously assess systemic and local lectin pathway protein concentrations in patients with various ILD. Our data suggests an involvement of pattern recognition receptors of the lectin complement pathway in sarcoidosis at the local level with significantly elevated lectin (MBL, ficolin-2, and ficolin-3) as well as complement split products (C4d and C5a) concentrations in the lungs of these patients. Additional analyses in a larger patient cohort are required to confirm or refute a potential effect of local and/or systemic ficolin-2 levels in IPF patients.

## Data Availability Statement

The raw data supporting the conclusion of this article will be made available by the authors, without undue reservation, to any qualified researcher.

## Ethics Statement

The studies involving human participants were reviewed and approved by the Ethical Review Board of the University Hospital of Basel (EKBB 05/06). The patients/participants provided their written informed consent to participate in this study. Written informed consent was obtained from the individual(s) for the publication of any potentially identifiable images or data included in this article.

## Author Contributions

MO and KH designed the study. MO and SV carried out all experiments. DS and MTa recruited all patients. MO, MTr, KH, and SV contributed to database design and data collection. SV drafted the manuscript and performed the statistical analysis. All authors were involved in the interpretation of data, critical revision of the manuscript for important intellectual content, and read and approved the final manuscript.

## Conflict of Interest

The authors declare that the research was conducted in the absence of any commercial or financial relationships that could be construed as a potential conflict of interest.
