# Supplementary material for: Local and Systemic Concentrations of Pattern Recognition Receptors of the Lectin Pathway of Complement in a Cohort of Patients With Interstitial Lung Diseases
Source: Front Immunol. 2020 Sep 23;11:562564. doi: 10.3389/fimmu.2020.562564 (PMC7546814; doi:10.3389/fimmu.2020.562564)
Supplement: Supplementary file 1 [file Table_1.DOCX]

# Supplementary Material

**Table S1** Spearman correlation between lung function parameters and lectin concentrations in the ILD-O group

|  | FVC | FEV1 | DLCOc |  |
| --- | --- | --- | --- | --- |
|  | Spearman’s rho  (P- value) | Spearman’s rho  (P-value) | Spearman’s rho  (P-value) |  |
| MBL |  |  |  |  |
| Plasma | -0.02 (0.89) | -0.08 (0.63) | 0.15 (0.38) |  |
| BALF | 0.18 (0.31) | 0.21 (0.23) | 0.26 (0.13) |  |
| Ficolin-2 |  |  |  |  |
| Plasma | 0.39 (**0.02**)* | 0.25 (0.16) | 0.28 (0.10) |  |
| BALF | 0.04 (0.82) | -0.13 (0.46) | -0.10 (0.58) |  |
| Ficolin-3 |  |  |  |  |
| Plasma | 0.03 (0.86) | 0.01 (0.97) | 0.29 (0.09) |  |
| BALF | 0.22 (0.20) | 0.07 (0.68) | 0.16 (0.35) |  |

MBL, mannose-binding lectine; BALF, bronchoalveolar lavage fluid; FVC, forced vital capacity, FEV1, forced expiratory volume in 1 second; DLCOc, diffusing capacity of the lung for carbon monoxide (adjusted for hemoglobin).

**Table S2** MBL2 allelic variants in cases and controls. A denotes the “wild type” allele, B the codon 54 allele, C the codon 57 allele and D the codon 52 allele.

|  | Controls | IPF | ILD-O | Sarcoidosis |
| --- | --- | --- | --- | --- |
| *MBL2* Exon 1 variants |  |  |  |  |
| *MBL2* A/B, n (%) |  |  |  |  |
| A/A | 27 **(**68**)** | 8 **(**80**)** | 26 **(**74**)** | 25 **(**71**)** |
| A/B | 11 (28) | 1 (10) | 7 (20) | 10 (29) |
| B/B | 2 (6) | 1 (10) | 2 (6) | 0 (0) |
| P-value* | Reference | 0.47 | 0.84 | 0.65 |
|  |  |  |  |  |
| *MBL2* A/C, n (%) |  |  |  |  |
| A/A | 39 (98) | 10 (100) | 32 (91) | 33 (94) |
| A/C | 1 (2) | 0 (0) | 3 (9) | 2 (6) |
| P-value* | Reference | 1 | 0.33 | 0.6 |
|  |  |  |  |  |
| *MBL2* A/D, n (%) |  |  |  |  |
| A/A | 31 (78) | 10 (100) | 29 (83) | 28 (80) |
| A/D | 9 (22) | 0 (0) | 4 (11) | 7 (20) |
| D/D | 0 (0) | 0 (0) | 2 (6) | 0 (0) |
| P-value* | Reference | 0.18 | 0.2 | 1 |
|  |  |  |  |  |
| *MBL2* promoter variants |  |  |  |  |
| *MBL2* X/Y, n (%) |  |  |  |  |
| Y/Y | 26 (65) | 4 (40) | 23 (66) | 27 (77) |
| Y/X | 11 (28) | 6 (60) | 11 (31) | 8 (23) |
| X/X | 3 (8) | 0 (0) | 1 (3) | 0 (0) |
| P-value* | Reference | 0.3 | 0.79 | 0.3 |

* Fisher’s exact test

**Table S3** *FCN2* allelic variants in cases and controls.

|  | Controls | IPF | ILD-O | Sarcoidosis |
| --- | --- | --- | --- | --- |
| FCN2 promoter variants |  |  |  |  |
| FCN2 -602, n (%) |  |  |  |  |
| G/G | 27 (68) | 7 (70) | 22 (63) | 23 (66) |
| G/A | 11 (28) | 3 (30) | 13 (37) | 11 (31) |
| A/A | 2 (5) | 0 (0) | 0 (0) | 1 (3) |
| P-value* | Reference | 1 | 0.50 | 0.92 |
|  |  |  |  |  |
| FCN2 -4, n (%) |  |  |  |  |
| A/A | 22 (55) | 5 (50) | 20 (57) | 18 (51) |
| A/G | 16 (40) | 3 (30) | 14 (40) | 16 (46) |
| G/G | 2 (5) | 2 (20) | 1 (3) | 1 (3) |
| P-value* | Reference | 0.30 | 1 | 0.92 |
|  |  |  |  |  |
| FCN2 exon variants |  |  |  |  |
| FCN2 +6359 |  |  |  |  |
| C/C | 19 (48) | 5 (50) | 17 (49) | 14 (40) |
| C/T | 19 (48) | 3 (30) | 16 (46) | 19 (54) |
| T/T | 2 (5) | 2 (20) | 2 (6) | 2 (6) |
| P-value* | Reference | 0.25 | 1 | 0.86 |
|  |  |  |  |  |
| FCN2 +6424, n (%) |  |  |  |  |
| G/G | 29 (72) | 8 (80) | 30 (86) | 27 (77) |
| G/T | 11 (28) | 2 (20) | 5 (14) | 8 (23) |
| P-value* | Reference | 1 | 0.26 | 0.79 |

*Fisher exact test

Estimation of epithelial lining fluid (ELF) volume:

$$Volume ELF \left( ml \right)= \frac{(Volume BALF \left( ml \right) x Urea BALF (mg/ml))}{Urea Plasma (mg/ml)}$$

Estimation of solute concentrations (MBL, ficolin-2, ficolin-3) in ELF:

$$Solute ELF (mg/ml) = \frac{(Solute BALF (mg/ml) x Volume BALF (ml))}{Volume ELF (ml)}$$

Volume BALF: volume of BALF recovered (in ml)

Urea BALF: concentration of urea in the recovered BALF (in mg/ml)

Urea Plasma: concentration of urea in the plasma (in mg/ml)

Solute BALF: concentration of lectins (MBL, ficolin-2, ficolin-3) in BALF (in mg/ml)
